# Anaesthetic Management of Distal Penile Hypospadias in a G6PD-Deficient Adolescent: Subarachnoid Block as a Safe Alternative

**DOI:** 10.7759/cureus.52998

**Published:** 2024-01-26

**Authors:** Inayat Garg, Gopal Jalwal, Jyoti Kanwat, Soumya Murmu

**Affiliations:** 1 Anaesthesiology and Critical Care, All India Institute of Medical Sciences, Bathinda, Bathinda, IND; 2 Anaesthesiology, All India Institute of Medical Sciences, Bathinda, Bathinda, IND

**Keywords:** antioxidant, dexmedetomidine, hemolysis, subarachanoid block (sab), glucose 6 phosphate dehydrogenase (g6pd)

## Abstract

Glucose-6-phosphate dehydrogenase (G6PD) deficiency is the most common X-linked recessive red blood cell disease in humans. The highest prevalence of G6PD deficiency is reported to be in Africa, Southern Europe, the Middle East, Southeast Asia, and the islands of the Central and South Pacific. G6PD deficiency causes acute hemolysis upon exposure to oxidative stress. Various stress factors that can cause hemolysis include infections, fever, sepsis, various foods such as fava beans, and various medications. In this report, we describe the case of a 13-year-old child who was diagnosed with G6PD deficiency in childhood but did not experience typical complications, such as hemolysis or jaundice. This child underwent Mathieu's flip-flap surgery for the treatment of distal penile hypospadias under spinal anesthesia and underwent the procedure uneventfully, with no hemolytic complications, malignant hyperthermia, or methemoglobinemia. Therefore, the main goals of our anesthesia management are to avoid various agents that cause hemolysis, use agents with antioxidant properties, reduce the stress of surgery through appropriate pain management, and monitor for signs of hemolysis. Therefore, in our case, subarachnoid blockade was found to be a safe and effective anesthetic technique compared with general anesthesia in the treatment of children with G6PD deficiency. Dexmedetomidine has antioxidant properties, maintains upper respiratory tract patency, and has sedative effect. Therefore, in our case, it was administered intravenously for perioperative management.

## Introduction

Glucose-6-phosphate dehydrogenase deficiency (G6PD) is an inherited X-chromosome-linked disease that affects approximately 400 million people [1], the majority of whom are men. G6PD is involved in the first step of the pentose phosphate pathway and produces antioxidants, nicotinamide adenine dinucleotide phosphate (NADPH) in red blood cells (RBCs). The most important role of NADPH in RBCs is the regeneration of reduced glutathione, which acts as an antioxidant and protects cells from oxidative damage [2]. In conditions of deficiency, most body tissues have other mechanisms to combat oxidative stress, but, for RBCs, this is the only way [3]. Therefore, patients with G6PD deficiency are susceptible to oxidative stress due to a lack of ability to protect RBCs from stressors, such as infections, certain drugs, fava beans, and sepsis.

WHO has classified the different G6PD variants based on the functional severity of G6PD enzyme deficiency and the severity of hemolysis. Class I G6PD deficiency is associated with chronic non-spherocytic hemolytic anemia. Class II variants have less than 10% enzyme activity, but no chronic nonspherocytic anemia. Class III mutants have 10-60% residual enzyme activity. Class IV mutants exhibit normal enzymatic activity [3,4].

Hemolysis in G6PD-deficient patients typically occurs within one to three days of exposure to oxidative stress and eventually resolves. Therefore, in the perioperative period, it is important to ensure that the patient has no signs of hemolysis, such as anemia, fatigue, tachycardia, abdominal pain, splenomegaly, hemoglobinuria, and jaundice, and, secondly, to ensure that it is important to avoid using medications that can cause oxidative stress such as paracetamol, benzocaine, and other drugs (e.h., sulfonamides, quinine, acetylsalicylic acid, methylene blue, isoflurane, sevoflurane, diazepam, and midazolam) [5]. On the other hand, studies have shown that dexmedetomidine is an anesthetic with antioxidant properties [6].

In this report, we demonstrate the successful use of subarachnoid blockade to provide safe and effective anesthesia and postoperative analgesia in patients with G6PD deficiency [7]. We also demonstrate the use of dexmedetomidine for intraoperative and peri-operative sedation and analgesia in patients with G6PD deficiency.

## Case presentation

**Figure 1:**
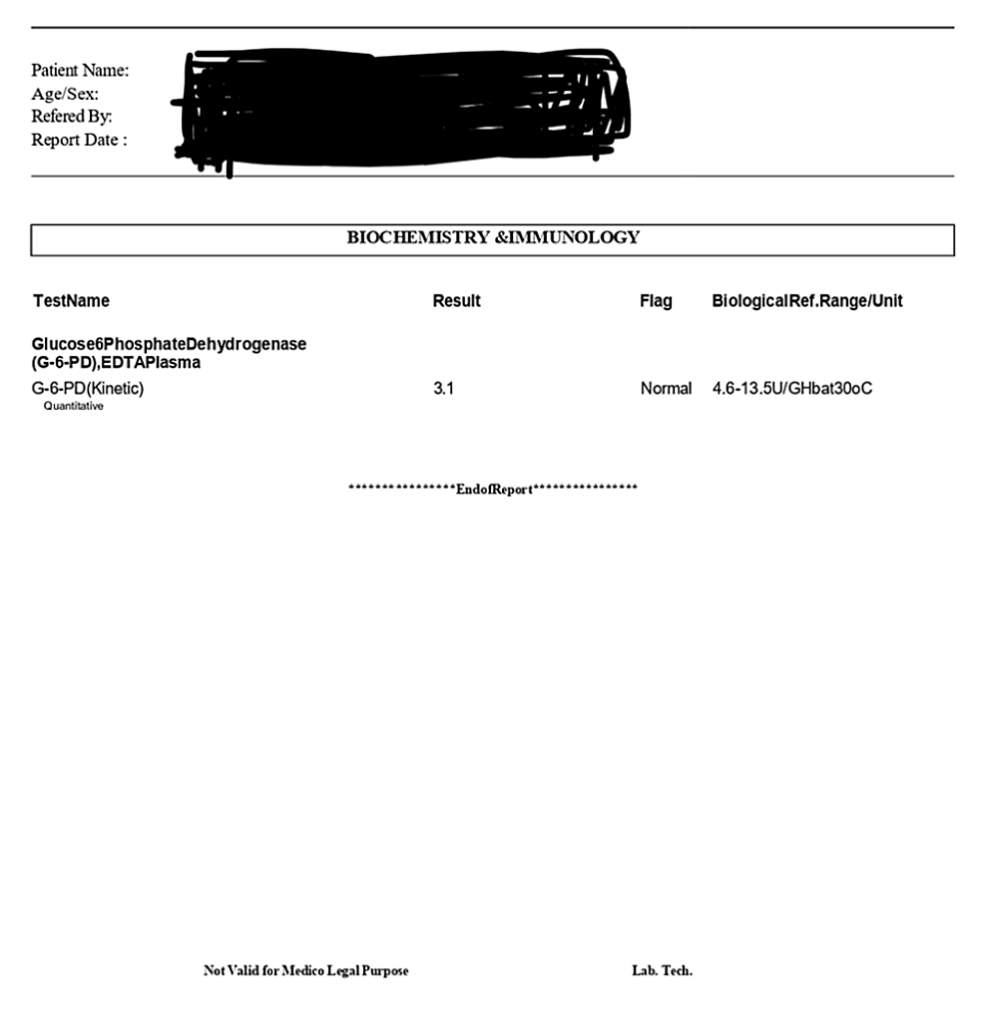
Diagnostic test of this patient for G6PD deficiency

A 13-year-old boy, weighing 65 kg, presented at the pediatric surgery clinic of All India Institute of Medical Sciences (AIIMS), Bathinda, with distal penile hypospadiasis. He was born by a vaginal route at 37 weeks of gestation, with a birth weight of 2,200 g. He was diagnosed with G6PD deficiency since childhood by the G6PD, ethylenediamine tetraacetic acid (EDTA) plasma test with a value of 3.1 (normal range is 4.6-13.5 U/G Hb at 30 °C) (depicted in Figure 1).

**Figure 2:**
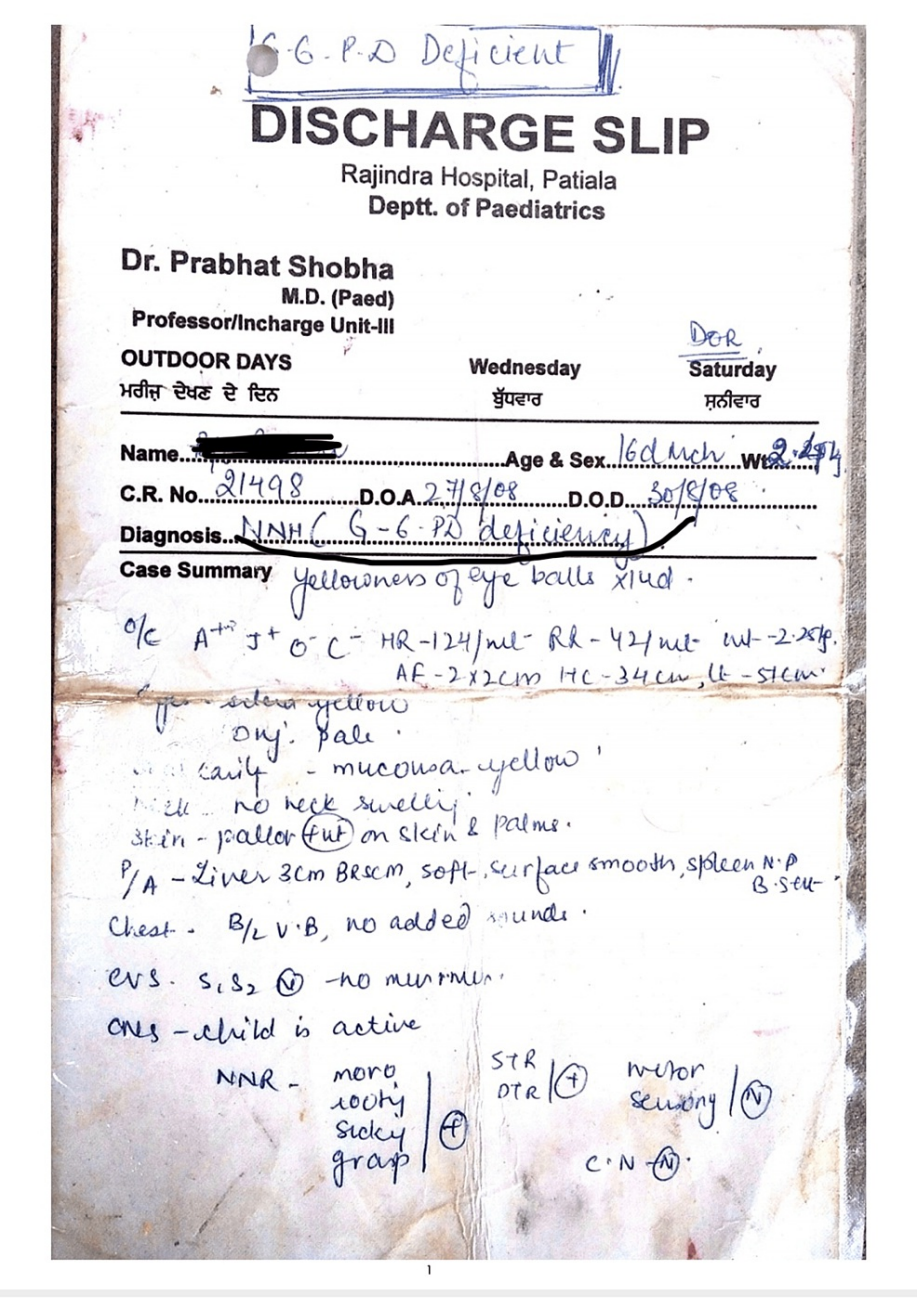
Additional image (evidence of G6PD deficiency)

However, no typical complications such as haemolysis, jaundice, or a history of multiple blood transfusions, were present. All routine preoperative investigations were within the normal limit (haemoglobin of 12.6 gm%, unconjugated bilirubin of 0.7 mg%, and reticulocyte count of 1%) (Figure 2).

This child was classified as ASA grade 1 according to the American Society of Anaesthesiologists and was scheduled for Mathieu’s flip-flap procedure. Informed and written consent for surgery and anaesthesia was obtained from the parents. After careful evaluation, it was decided that the anesthesia technique for this procedure and the patient would be spinal anesthesia.

In the operating room, intravenous access was secured. Standard intraoperative monitoring was applied, including electrocardiogram, non-invasive blood pressure, pulse oximetry, and temperature. Dexmedetomidine infusion at a rate of 0.5 mcg/kg/hour was also started via continuous intravenous dosing rather than a loading dose. Oxygen was administered via nasal cannula at the rate of 3 L/minute. The patient was positioned appropriately, and landmarks for the subarachnoid block were identified. Following an aseptic protocol and after confirmation by the free flow of cerebrospinal fluid, a subarachnoid block was given using 0.4 mg/kg body weight (2.8 mL) of 0.5% hyperbaric bupivacaine with 10 μg of clonidine as an adjunct at L4/L5 interspinal space using 25 gauge Quincke Babcock spinal needle. This was given as an effective alternative to the general anaesthesia technique. The temperature of the operation theatre was also maintained at around 36.8-37.4 degrees for the entire duration of the surgery, which was two hours.

The following measures were taken to prevent precipitation of hemolysis in this child; the methylated spirit was avoided in the solutions used to prepare the skin, local site infiltration with lidocaine was not done, hypothermia was prevented by switching off the air-conditioning and using a bear hugger, and hydration was done intraoperatively with 15-20 mL/kg body weight of intravenous crystalloid fluids. The intraoperative and postoperative course was uneventful as no haemolytic complications, malignant hyperthermia, or methaemoglobinaemia occurred. After the procedure, the patient was observed in the PACU for one hour and shifted to the paediatric ward. The patient was discharged after the third postoperative day after the complete blood count was normal. The patient was followed up in paediatric surgery outpatient department one week and one month later, which was found to be uneventful.

## Discussion

The main goal of our anaesthetic management is to avoid various drugs leading to hemolysis, use drugs with antioxidant action, reduce surgical stress by adequate pain management, monitor for any signs of haemolysis, and ensure adequate preparations to manage haemolysis if it occurs [7]. As the procedure was carried out below the level of the umbilicus, subarachnoid block (SAB) was the preferred anaesthetic technique here. It avoided multiple drugs used in general anaesthesia carrying the risk of haemolysis (Table 1). SAB also plays a role in post-operative pain management, thus reducing the need for additional analgesics to an extent. In local anaesthetics, lidocaine and prilocaine are not recommended, but bupivacaine has been mentioned in studies as a safe and adequate alternative [8,9]. In in vitro studies, various drugs, such as isoflurane, sevoflurane, diazepam, and midazolam, have shown an inhibitory effect on G6PD activity; thus, these drugs should be avoided as they may increase the severity of hemolysis [10].

**Table 1:** Drugs and chemicals associated with hemolysis in G6PD deficiency Class I - severely deficient, chronic hemolytic anaemia Class II - 1-10% residual activity, but no chronic nonspherocytic anemia Class III - 10-60% residual activity

Unsafe for Classes I, II, and III	Safe for Classes II and III
Acetanilid	Phenytoin
Dapsone	Procainamide
Furazolidone	Quinidin
Methylene blue	Quinine
Nalidixic acid	Streptomycin
Niridazole	Pyrimethamine
Nitrofurantoin	Acetaminophen
Phenazopyridine	Aminopyrine
Phenylhydrazine	Ascorbic acid
Primaquine	Aspirin
Sulfacetamide	Isoniazid
Paraaminobenzoic acid	L-Dopa
Sulfamethoxazole	Menadione
Trinitrotoluene	Colchicine

Dexmedetomidine has antioxidant, anti-inflammatory, and sedative effects. It is less likely to cause respiratory suppression and maintains upper airway patency in children; thus, it is reported as a useful sedative in infants and children [11]. In our study, a continuous infusion dose was given, and adequate peri and intraoperative hydration was done; thus, no significant changes in intraoperative blood pressures were seen.

Typically, drug-induced haemolysis occurs at 24-72  hours, while symptoms of anaemia appear by seven days after haemolysis in G6PD deficiency patients. Therefore, it is important to pay particular attention to signs and symptoms of hemolysis in the postoperative period for at least a week. In the above case, a normal complete blood count on postoperative day three was sufficient laboratory confirmation for the child’s recovery and discharge.

## Conclusions

This case report suggests the use of subarachnoid block as a safe and effective alternative to general anaesthesia in children with G6PD deficiency for surgical procedures below the umbilical level in whom the drug choice may be limited. Drugs with antioxidant effects can be employed to aid in intra- and perioperative anaesthesia for the safe and effective management of pain and anxiety in these patients.
